# Expression Quantitative Trait Loci (eQTLs) Associated with Retrotransposons Demonstrate their Modulatory Effect on the Transcriptome

**DOI:** 10.3390/ijms22126319

**Published:** 2021-06-12

**Authors:** Sulev Koks, Abigail L. Pfaff, Vivien J. Bubb, John P. Quinn

**Affiliations:** 1 Perron Institute for Neurological and Translational Science, Perth, WA 6009, Australia; abigail.pfaff@uwa.edu.au; 2 Centre for Molecular Medicine and Innovative Therapeutics, Murdoch University, Perth, WA 6150, Australia; 3 Department of Pharmacology and Therapeutics, Institute of Systems, Molecular and Integrative Biology, University of Liverpool, Liverpool L69 3BX, UK; jillbubb@liverpool.ac.uk (V.J.B.); jquinn@liverpool.ac.uk (J.P.Q.)

**Keywords:** transposable element, transcriptome, whole genome sequencing, whole transcriptome analysis, RNA-seq, gene expression regulation, expression quantitative trait loci, HLA, WFS1

## Abstract

Transposable elements (TEs) are repetitive elements that belong to a variety of functional classes and have an important role in shaping genome evolution. Around 50% of the human genome contains TEs, and they have been termed the “dark matter” of the genome because relatively little is known about their function. While TEs have been shown to participate in aberrant gene regulation and the pathogenesis of diseases, only a few studies have explored the systemic effect of TEs on gene expression. In the present study, we analysed whole genome sequences and blood whole transcriptome data from 570 individuals within the Parkinson’s Progressive Markers Initiative (PPMI) cohort to identify expression quantitative trait loci (eQTL) regulating genome-wide gene expression associated with TEs. We identified 2132 reference TEs that were polymorphic for their presence or absence in our study cohort. The presence or absence of the TE element could change the expression of the gene or gene clusters from zero to tens of thousands of copies of RNA. The main finding is that many TEs possess very strong regulatory effects, and they have the potential to modulate large genetic networks with hundreds of target genes over the genome. We illustrate the plethora of regulatory mechanisms using examples of their action at the *HLA* gene cluster and data showing different TEs’ convergence to modulate *WFS1* gene expression. In conclusion, the presence or absence of polymorphisms of TEs has an eminent genome-wide regulatory function with large effect size at the level of the whole transcriptome. The role of TEs in explaining, in part, the missing heritability for complex traits is convincing and should be considered.

## 1. Introduction

Eukaryotic genomes are replete with interspersed repetitive elements originating from transposable elements (TEs) [1]. TEs can transpose within the genome and generate de novo mutations or re-arrangements. Depending on the structure, sequence similarities and replicative mechanism, TEs are classified into variable subcategories [2]. Class I are the retroelements that use RNA intermediates to replicate, they are reverse transcribed and inserted into a new genomic location [3]. There are two large subclasses of retroelements, long terminal repeat (LTR) and non-LTR retrotransposons. [4]. LTR elements, which include endogenous retroviruses, have quite well-preserved viral structure flanked by LTRs at both ends, a group-specific antigen (Gag), reverse transcriptase (RT) and a retroviral envelope protein (Env) [4]. Non-LTR retrotransposons consist of a long-interspersed element (LINE), short-interspersed element (SINE) and SINE-VNTR-*Alu* (SVA) families. Class II elements are DNA transposons that transpose directly from DNA to DNA and have characteristic short terminal inverted repeats at both ends [3]. They integrate by cutting out from the original position and inserting themselves into a new locus and are no longer able to transpose in the human genome [3]. This study will focus on the non-LTR retrotransposons.

The new copy of the TE is identical to its origin and starts to diverge from the initial sequence; therefore, during evolution, families of elements have formed with sequence similarities [5]. This process induces the diversification of the original sequences and enables classification between the younger and older elements. Over time, elements accumulate mutations and eventually lose their ability to transpose [3]. The younger TEs have not generated sufficient mutations to incapacitate their ability to transpose and therefore, can still actively mobilise [6]. These actively transposing elements in the human genome consist of only a small number of elements, and they belong to the LINE-1 (L1), *Alu* and SVA families of the non-LTR retrotransposons. L1s are the only autonomous retrotransposons and encode two proteins, ORF1p, an RNA binding protein, and ORF2p with reverse transcriptase and endonuclease activity, required for mobilisation. *Alus*, originating from the fusion of two monomers derived from the 7SL RNA gene, and SVAs, a hominid-specific composite element, utilise the proteins encoded by L1 for their mobilisation [7]. Both old and young elements still retain the same functional consequences for gene regulation from the original mobilisation event. For mobilisation specifically, TEs, such as L1, can retain functional autonomous promoters, which allow them to remain transcriptionally active and independent of cellular promoters and allows them to regulate the expression of genes or larger genetic networks utilizing different mechanisms [8,9]. Thus mechanistically, newly inserted TEs contain *cis*-acting sequences, such as promoters or splice sites, and therefore have the potential to regulate host gene expression at their site of insertion. All this leads to the conclusion that TEs generate insertional polymorphic changes in the genome through mobilisation that may become active regulatory domains. 

Insertions of TE elements are responsible for a variety of genetic diseases. The most remarkable example is the presence of an SVA insertion in an intron of the *TAF1* gene causing the decreased expression of *TAF1* by intron retention, leading to X-linked dystonia-parkinsonism [10]. There are many examples demonstrating the association and/or role of TEs in human diseases [9], including haemophilia A and B, Duchenne muscular dystrophy, Apert syndrome, cystic fibrosis and breast cancer [9,11]. Our group has described the extra burden of L1 activity as associated with PD [12]. We have also described the increased expression of TE elements in different diseases [13,14,15]. These data indicate that TEs have an important impact on the inheritance or progression of diseases. This most likely reflects the ability of TEs to add an additional layer of complexity to our normal physiology through, in part, modification of the transcriptome. Therefore, analysis of presence/absence polymorphisms, termed retrotransposon insertional polymorphisms (RIP) of TEs, will provide additional information about the heritability of traits. Single nucleotide polymorphism (SNP)-based genome-wide association studies have proven very insightful, however, in polygenic diseases can be limited in the genetic factors identified, leading to the “missing heritability” problem often observed in such diseases. As RIPs are very frequent in the human genome, they could provide additional information about genetic variation that remains hidden when using only SNPs. Moreover, TEs have been shown to regulate gene expression and networks of functionally linked genes [16,17]. 

In this study, we analysed the variation of TEs using whole genome sequence data to identify reference RIPs, i.e., TEs known to be RIPs in reference to the human genome, belonging to the L1, *Alu* and SVA families, and associated their polymorphism with gene expression using blood whole transcriptome data within the Parkinson’s Progression Markers Initiative (PPMI) cohort. Our goal was to identify the eQTLs for TEs and to analyse their effect size. As we focused on the L1, *Alu* repeats and SVAs, our data are presented separately for these three families. Our analysis identified significant and large regulatory effects on the whole transcriptome scale, and this finding can provide functional explanations to genetic disease-causing mechanisms.

## 2. Results

### 2.1. General eQTL Effects of the TEs 

**Figure 1:**
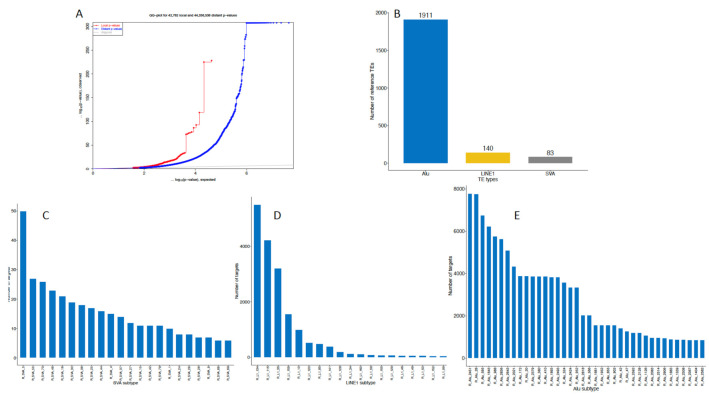
Summary statistical information about the analysed transposable elements (TEs). (**A**) is a Q-Q plot of identified p-values from eQTL analysis; (**B**) is a plot for all TE categories we analysed in our study. (**C**–**E**) are separate density plots for the eQTLs (targets) for SVA, L1 and *Alu* repeats.

Using whole genome sequencing data, we identified 2,132 reference TEs that were polymorphic in our study cohort (Figure 1A). Among these TEs, we had 1911 *Alu* repeats, 140 L1s and 81 SVAs that were numbered according to their position in the human genome (Figure 1B). All these genetic variants were used for the eQTL analysis on blood-derived whole transcriptome data from the same subjects. We discovered 189,941 genetic loci that were differentially regulated by TEs at genome-wide significance level with the FDR below 0.05 (Figure 1A). Of these genes 525 were *cis*-regulated, and 189,416 were *trans*-regulated (Supplementary Table S1). A single TE can demonstrate both modulation of a single target gene or multiple genes. Different families of TEs and number of genes modulated are presented in Figure 1. The largest number of genes modified by an individual TE by specific class was 50 targets for SVA-5 (Figure 1C), 5,497 targets for L1-134 (Figure 1D) and for *Alu*-2451 element 7,781 targets (Figure 1E) over the genome.

**Figure 2:**
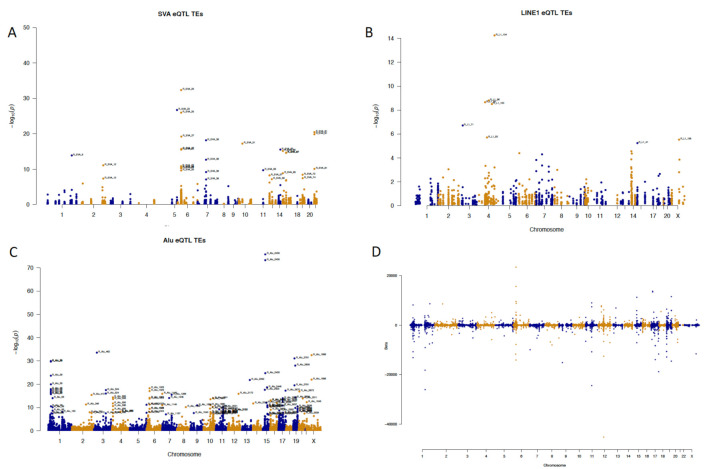
(**A**–**C**) Manhattan plots of SVA, L1 and *Alu* repeats illustrating the FDR values and locations of the target genes. (**D**) Manhattan plot showing effect sizes (beta values) of all TEs and the locations of their respective target genes/eQTLs.

The identified TE elements had diverse but clearly localised effects by targeting certain genomic regions with FDR values up to the 10^−50^ to 10^−200^ range. Each group of TEs behaved differently and had its own characteristic effects. For example, SVAs had the lowest FDR values for chromosome 6 (Figure 2A); L1s had statistically the most significant signals in chromosome 4 (Figure 2B), and *Alu* elements had signals with the lowest FDR values in chromosome 15 (Figure 2C). This indicates that eQTL targets are TE-element-specific, and each of the elements may prefer its own cluster of targets. The statistical significance measured with *p*-values or FDR values may sometimes not be sufficient to describe the impact of the analytical results, and adding the effect size is important to understand the magnitude of the measured effects. We used beta (slope coefficient) to measure the magnitude of the effect induced by each of the TEs. The beta values for all TEs are shown in Figure 2D. The highest beta coefficient for the SVA elements was 24,858, and the lowest was −3900 (Figure 2D and Supplementary Table S2). For L1 elements, the highest beta value was 1625, and the lowest was –178,823 (Figure 2D and Supplementary Table S3). In the case of the *Alu* elements, the beta values were 11,288 for the maximal value and −2400,511 for the minimal value (Figure 2D and Supplementary Table S4). The tables describe beta values as minimum, maximum and mean values to exhibit an overview of the effect size each element had in our study.

**Figure 3:**
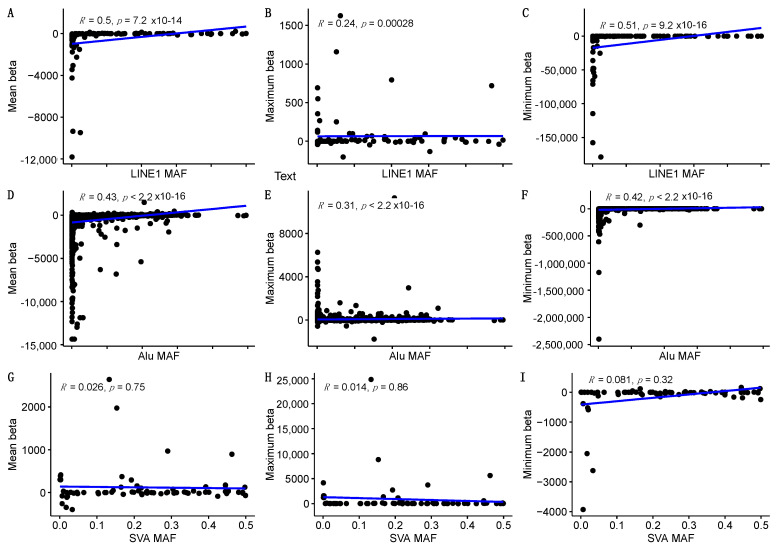
Correlation analysis between allele frequencies and beta values for L1, *Alu* and SVA. **A**, **B** and **C** show correlation between LINE1 MAF and its effect size and direction. **D**, **E** and **F** show correlation between Alu MAF and its effect size and direction. Panels **G**, **H** and **I** show correlation between SVA MAF and its effect size and direction. Mean beta is an average effect size for a specific TE, maximum beta shows activating effects for specific TE and minimum beta indicates suppressive regulatory effect. The effect of LINE1 and Alu elements had clear MAF dependent correlation. SVA effect did not correlate with MAF.

We next analysed the frequency dependency of the effect size for the TEs and performed correlation analysis between the minor allele frequency (MAF) and beta values of the respective elements (Figure 3). As the beta values had a large range, we divided the effect sizes into mean, maximum and minimum values. Minimum values represent repression of the genes; maximum values represent enhancement, and means give general effect size for the group of TEs. SVAs didn’t have any significant correlation with the allele frequency of the element (Figure 3G–I). 

In contrast, both L1s and *Alu* repeats had a frequency-dependent effect with highly significant correlation (Figure 3A–F). It is important to stress here that the MAF-dependent effect was significant for both enhancement (maximum beta) and repression (minimum beta) effects of TEs on gene expression. Correlation coefficients for L1 and *Alu* elements were all positive, meaning that the smaller the MAF, the more negative was the effect, and the larger the MAF, the more positive the effect was (Figure 3A–F).

### 2.2. Element-Specific eQTL Effects in the Genome 

In the next step, we focused on more specific effects of each subgroup of elements. As evident from the Supplementary Tables S2–S4, each of the TE groups seem to have different targets and different effect profiles.

**Figure 4:**
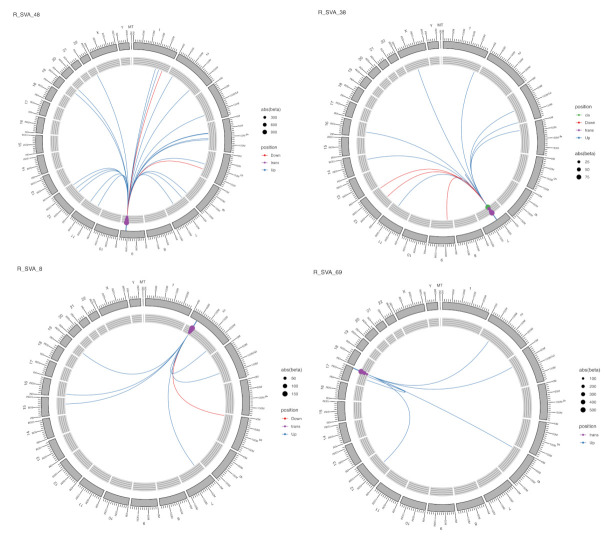
Circos plots showing the targets and effects for four different SVAs. Blue line is for up-regulation; red line is for down-regulation of the gene. Dot size is beta value; dot colour is *trans* or *cis* effect.

The distinctive feature for SVAs is that they usually have gene-activating or up-regulating effect, but this effect depends on the targets rather than on the TE itself. SVAs had by far the largest up-regulating effect for the genes they regulate (Supplementary Table S1), for example SVA-25 and SVA-27 on the HLA-B and HLA-C genes. HLA-B was also regulated by SVA-26, which is adjacent to SVA-25 and SVA-27. This is typical in our results, that TEs usually have more than one target gene as only 19 out of 71 statistically significant SVAs regulated single targets (Supplementary Table S2). The majority of SVAs had two to ten targets, and 15 SVAs had more than ten targets; for example SVA-5 had 50 targets in the genome. Figure 4 illustrates four distinct SVAs on different chromosomes and demonstrates the range of affects we observed in their ability to modulate gene expression. SVA-48 on chromosome 9 had 23 targets, all *trans* regulated; expression was enhanced for most of the targets; however, two were repressed (Figure 4). SVA-38 on chromosome 7 had 17 targets; one was *cis*-regulated, and the rest were *trans*-regulated; three were repressed, and the rest of the target’s expression was enhanced. SVA-8 on chromosome 2 had seven targets, all in *trans*, one repressed and six enhanced. SVA-69 on chromosome 17 had six targets whose expression was enhanced. 

**Figure 5:**
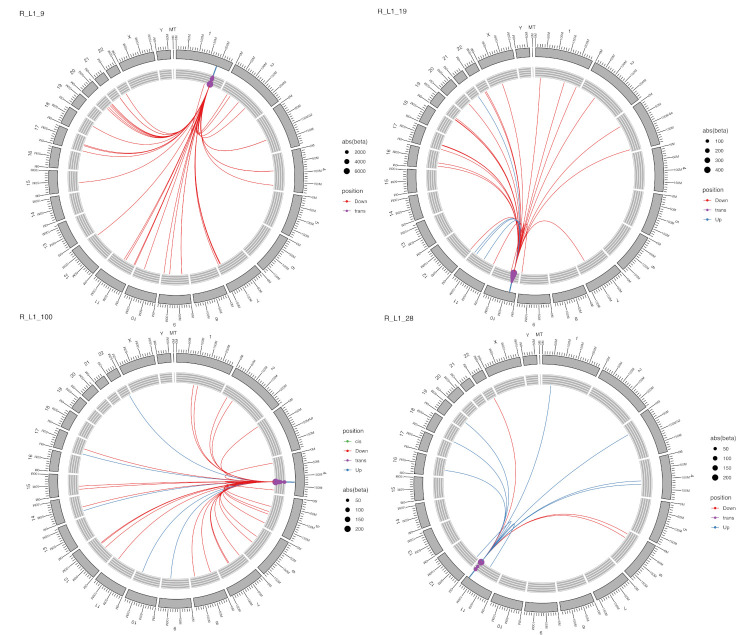
Circos plots showing the targets and effects for four different L1s. Blue line is for up-regulation; red line is for down-regulation of the gene. Dot size is beta value; dot colour is *trans* or *cis* effect.

L1 elements contribute to 17% of the human genome and are the only autonomous TE [12]. Out of all studied L1 elements, 107 demonstrated statistically significant eQTL effects. Of these, only 13 of them had a single target locus; 51 had 2–10 loci, and 43 L1 elements had more than 10 loci with quantitative regulatory effect. For example, L1-134 had 5497 loci under its control; L1-115 had 4231 quantitative loci (Supplementary Table S3). The regulatory effect direction was quite diverse. Some effects of L1 are illustrated in Figure 5, in which L1-9 on chromosome 1 had 37 targets all repressed in *trans* with very large beta values. L1-19 on chromosome 10 had 27 targets in *trans* position with four enhancing effects. L1-100 on chromosome 4 had 32 targets, one in *cis* position and five targets under activating influence. L1-28 in chromosome 11 had 12 targets with mostly up-regulating effects, all *trans* and three down-regulating influence (Figure 5).

**Figure 6:**
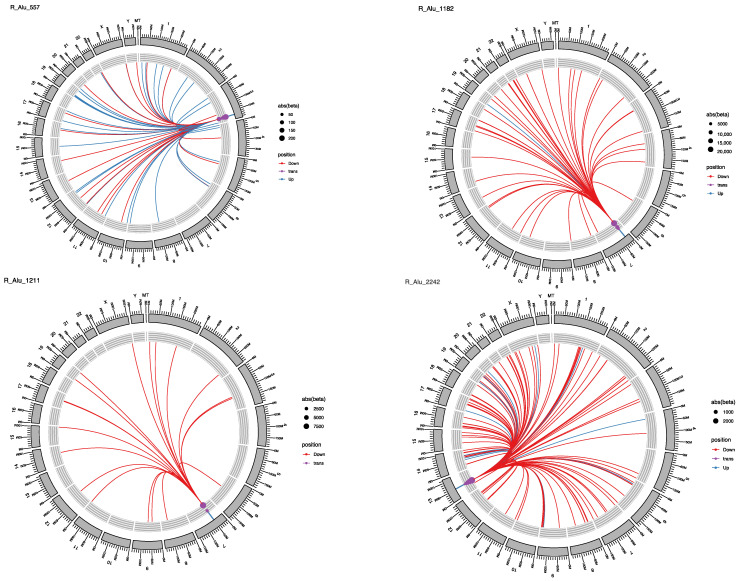
Circos plots showing the targets and effects for four different *Alus*. Blue line is for up-regulation; red line is for down-regulation of the gene. Dot size is beta value; dot colour is *trans* or *cis* effect.

The analysis of *Alu* repeats identified 1638 statistically significant eQTLs. Of these *Alu* elements, 143 had only a single eQTL target; 722 had two to ten targets, and 773 had more than 10. The repeat Alu-2451 had 7781 targeted eQTLs, and Alu-28 had 7775 eQTL loci (Supplementary Table S4). *Alu* repeats mostly had a repressive effect on the eQTL targets and demonstrated the largest repressive effects (the smallest beta values) of the three classes, L1, SVA and *Alu,* analysed. The lowest beta value for *Alu* repeats was −2400,511 (Supplementary Table S1 and Supplementary Table S4). Figure 6 illustrates four different *Alu* repeats with diverse profiles of eQTLs. Alu-557 had 56 eQTL in *trans* position, half of them up-regulating and half with down-regulating effects, as is shown by the almost equal blue and red lines in the circos plot. Alu-1182 had 51 eQTL targets, all in *trans* position and all except one with down-regulating activity. Alu-1211 had 19 targets, all of which were in *trans* position and had a supressing effect on gene expression. And finally, Alu- 2242 had 118 eQTL targets, all in *trans* position, 12 activating and 106 with supressing effects (Figure 6).

### 2.3. Locus-Specific eQTL Effects of the Selected TEs

We next analysed the locus-specific effects for some TEs in greater detail. This would delineate more specific changes that TEs can induce in their eQTL targets. 

**Figure 7:**
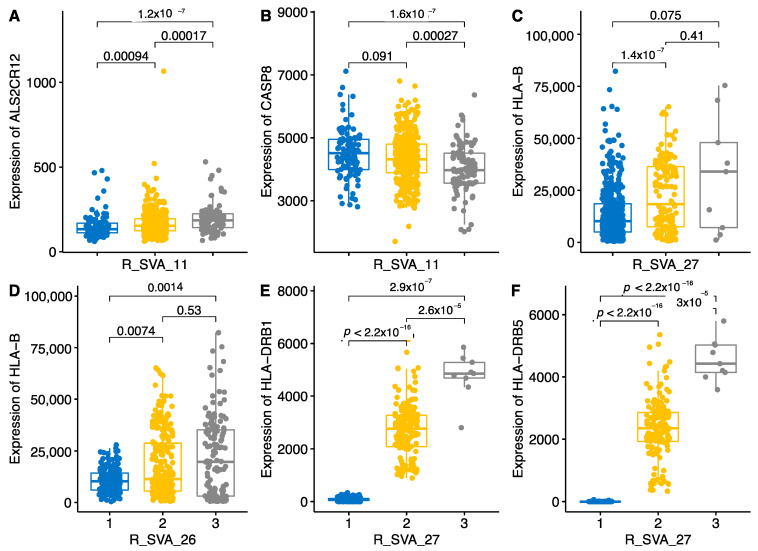
Boxplot of six different genes influenced by SVAs. Genotypes of SVAs are shown as number 1 for AA, 2 for PA and 3 for PP. Numbers and lines above the bar show *p*-values of the pairwise comparison. *p*-values are from the Wilcoxon pairwise comparison. Panels **A**–**F** indicate six genes and their expression dependence on the SVA genotype, Panel B shows dose-dependent downregulating effect of SVA-11.

SVA-11, which is located in an intron of *CASP8*, had statistically significant and copy-number-dependent eQTL effect for the genes *ALS2CR12* (*FLACC1*) and *CASP8*, two tail-to-tail adjacent genes. The presence of either one (PA) or two alleles/copies (PP) significantly up-regulated *ALS2CR12* expression (Figure 7A). The effect on *CASP8* is the opposite; it is downregulated by the presence of one or two alleles (Figure 7B). The presence of one or two copies of SVA-27 significantly upregulated HLA-B, HLA-DRB1 and HLA-DRB5 (Figure 7C,E,F). SVA-26 significantly upregulated HLA-B (Figure 7D).

**Figure 8:**
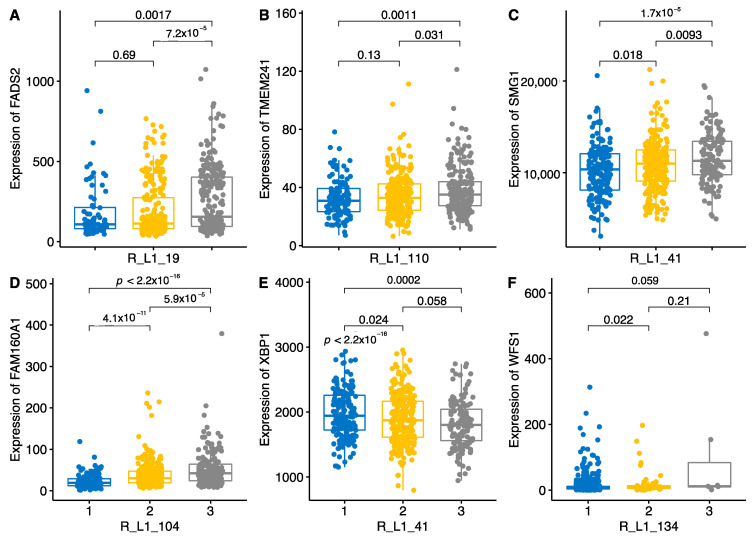
Boxplot of six different genes influenced by L1s. Genotypes of L1s are shown as number 1 for AA, 2 for PA and 3 for PP. Numbers and lines above the bar show *p*-values of the pairwise comparison. *p*-values are from the Wilcoxon pairwise comparison. Panels **A**–**F** show different regulatory effects of variable L1s. E panel shows dose-dependent downregulating effect.

Like the SVAs, L1s had clear copy-number-dependent effect on their target genes (Figure 8). For instance, the presence of L1-19 significantly up-regulated the *FADS2* gene (Figure 8A), one of the many targets for this L1 (Figure 5). L1-110 and L1-104 are other examples wherein the presence of the L1 element induces gene expression in an additive manner. L1-41 had gene expression activating and suppressing effects, depending on the target. The *SMG1* gene in *cis* position was significantly upregulated by the presence of the L1-41, whereas the *XBP1* gene in *trans* position was significantly down-regulated by the presence of L1-41 (Figure 8C,E). The *XBP1* gene is involved in the ER stress response and acts as a transcription factor to initiate the unfolded protein response (UPR). As *XBP1* has functional interaction with another ER-stress-related gene, *WFS1*, we also tested if *WFS1* gene is activated by any of the TEs or L1 elements [18]. We identified 14 TEs that regulate the expression of *WFS1*, three L1 and 11 *Alu* repeats regulating its expression, and only one L1, L1-134, had an activating effect that was dependent on the copy number of the L1-134 element (Figure 8F).

**Figure 9:**
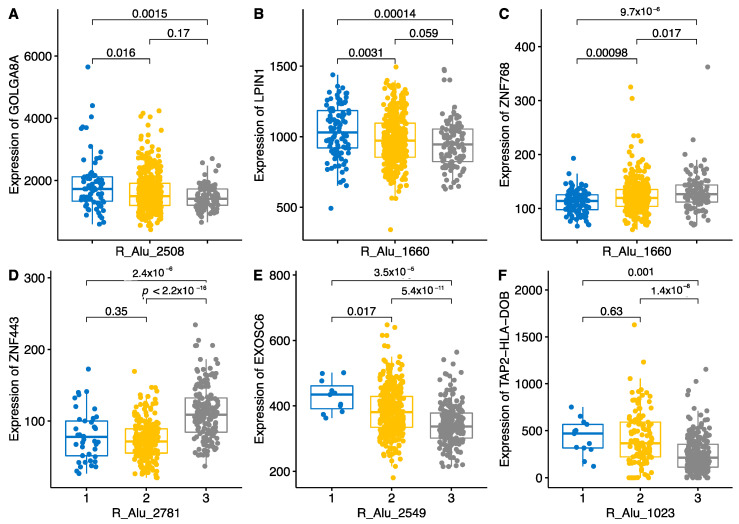
Boxplot of six different genes influenced by Alu repeats. Genotypes of Alus are shown as number 1 for AA, 2 for PA and 3 for PP. Numbers and lines above the bar show *p*-values of the pairwise comparison. *p*-values are from the Wilcoxon pairwise comparison. Panels **A**–**F** show variable regulation of genes by Alu elements. **A**, **B**, **E** and **F** show dose-dependent downregulating effect.

To illustrate similar copy-number-dependent patterns for *Alu* repeats, we choose a set of these elements and their targets (Figure 9). Alu-2508 down-regulated *GOLGA8A* dose-dependently. For Alu-1660, we used *LIPIN1* and *ZNF768* to illustrate the down-regulating and up-regulating effect of the element (Figure 9B,C). *Alu* repeats predominantly had a suppressing effect on gene expression, with a few exceptions. *ZNF768* is one of them; Alu-1660 had a statistically significant dose-dependent activating effect on it.

Another *Alu* repeat, Alu-2781, significantly stimulated the expression of *ZNF443*, but in general, *Alu* repeats had a strong down-regulating effect on, for example, the *EXOSC6* and *TAP2-HLA-DOB* genes. *TAP2-HLA-DOB* is a newly described readthrough gene (ENSG00000250264) that combines the *HLA-DOB* and *TAP2* genes (Figure 9F). This transcript had low expression in subjects with two copies of Alu-1023, and it was highly expressed in subjects without any copies of Alu-1023. We have described this transcript-specific regulation also in our previous publication in relation to *APOE4* haplotypes and *TOMM40* transcripts [19].

**Figure 10:**
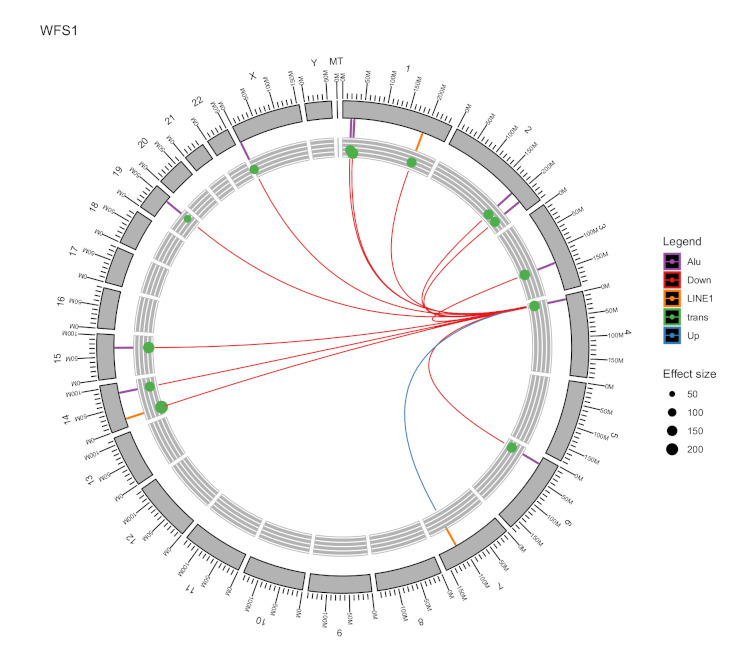
Circos plot of the TEs regulating the *WFS1* gene. *Alu* and L1 are indicated as purple or yellow bars respectively. Green dots indicate trans-regulating effects. Dot size illustrates beta value. Colour of the line indicates up-regulation (blue) or down-regulation (red).

Based on these findings and our previous interest in the ER-stress-regulated pathways, we next analysed the TE-based regulation of the *WFS1* gene, which is involved in neurodegenerative and mood disorders linked to endocrine pathologies and altered transcriptomic profiles [20,21,22]. We specifically looked for the statistically significant TEs that were associated with *WFS1* expression and identified 14 TEs (3 L1 and 11 *Alus*) regulating the expression of *WFS1* (Figure 10), and only one L1, L1-134, had an activating effect that was dependent on the copy number of the L1-134 element (Figure 8F). Thus, this illustrates the situation wherein a single target gene is under control of several different TEs. Interestingly, only one TE had an upregulating effect, and all others had downregulating effects. The *WFS1* gene is an example of how TEs can regulate genes and participate in gene-network regulations. Considering the role of *WFS1* gene in neurodegeneration, it is remarkable how tightly one gene is regulated by several different TEs. 

## 3. Discussion

A major outcome of our study is the description of the quantitative expression loci of non-LTR retrotransposons and the identification of their eminent effect on the regulation of gene expression. Our study focused on reference genome RIPs and demonstrated the huge impact this type of variation could have on genome wide transcription [23,24,25,26]. Gene expression profiling can be pathway-specific or whole-transcriptome-wide, depending on the hypothesis and goals of the studies. Therefore, it is important to consider the transcriptional impact of genomic variation that is due to TEs (RIPs). The present study also highlighted that this transcriptional regulation is not only restricted to the *cis* position.

Several previous studies have similarly identified the impact of TEs on gene expression [27,28]. Using the 1000 genome project data of 445 individuals from different populations, TEs were called and analysed in correlation with the RNA-seq data from the Epstein–Barr virus transformed B-lymphocytes [28]. RIPs were shown to have population-specific and cell-type-specific regulatory effect on the transcriptome. Similar to our analysis, that study identified a greater number of *trans* than *cis* positional regulators among TEs. Similarly to our study, they identified that many TEs have converging effects on single targets, and therefore, genes are under the control of several TEs simultaneously [28]. Our analysis models were identical to the study by Wang et al. (2017) [28], and our results were similar, providing confidence that the results of these different studies are comparable. In our study, we extended the data on the differences that TE families have and on the effect size of these elements.

Our main finding is the differential effect of the TE subtypes. SVAs had a clearly stronger activating effect on gene expression with the largest beta values of 24,858. The lowest value was −3900, showing a predominant activating effect of SVAs. At the same time, L1 elements and *Alu* repeats had a profound repressive effect on the target loci, evidenced by the large negative beta values. One conclusion here is that SVA insertions are therefore usually activating, while *Alu* and L1 insertions are more often repressive. Further the magnitude of the beta values we reached in our study were in the range of tens of thousands and, in some cases, even hundreds of thousands, indicating the profound regulatory impact of TEs. That large effect size has not previously been described for TEs to our knowledge and clearly indicates the importance of these elements in genomic regulatory networks. We also performed correlation analysis between the allele frequencies and effect sizes to identify possible evolutionary selection on the elements. In the case of L1 and *Alu* repeats, we observed significant correlation between effect size and MAF, however, for SVAs, no such correlation was detected, which could be explained by evolutionary differences.

Genome-wide transcriptome analysis supplies a useful tool for understanding the basis of multifactorial diseases as it offers a great opportunity to identify both biomarkers and a better understanding of disease mechanisms [23,25,29]. Profiling of the transcriptome is easy to perform and often supplies insight into pathological processes [30,31]. Our data indicates that it is important to consider, not only the SNP based genotype profile of the patients, but also the potential impact of the variation due to RIPs when analysing transcriptomes. This data should be more accessible in the future with increased access to WGS. As seen in the present study, RIPs may significantly change gene-expression signals. RIPs can be considered analogous to the genomic background effect described so commonly in studies of transgenic mice. In these rodent models, differential affects are often seen that are dependent on genetic background. We have shown the impact of these using transgenic models to describe the congenic-footprint effect that can far too easily induce false-positive profiles in animal models [18,32]. Perhaps it is appropriate to consider RIPs in the context of such transcriptome profiling.

In addition to RIPs modifying genomic structure to alter gene expression directly, TEs themselves can be expressed, and this expression affects many multifactorial diseases and conditions [13,14,33,34,35,36]. For example, L1 expression can increase DNA damage via the expression of the encoded endonuclease, and researchers have described the expressional activation of TEs and HERVs in cases of different neurological diseases [37]. TE transcription is also related to the ageing process [38]. The tools for TE transcriptional profiling are constantly evolving, but they still need to be developed further to capture the repetitive nature of TEs, a nature that makes them difficult to call correctly with short-read WGS data [3,39]. In addition, the transcription of TEs can be a highly diverse process, combining full-length transcripts with variably truncated transcripts that make the profiling even more challenging [3].

In addition to RIP variation, TEs have an added layer of polymorphism within the TE itself. For example, SVAs are composite elements containing SINE-VNTR-*Alu* domains and are a relatively recent family of TEs to enter the genome [40,41]. These elements are polymorphic in multiple domains, including the variable number tandem repeat (VNTRs) element and the CT hexamer repeat in one of the termini. The latter variation affects age of onset in XDP [42]. VNTRs more generally have been studied extensively as both biomarkers and functional elements in the context of complex genetics for many years, and these elements have been shown to have tissue-specific and stimulus-inducible regulatory properties, which are modified further by polymorphism in the VNTR itself [43]. We would expect such polymorphism to play a role in the variable function of the RIP on gene expression in that it is not solely due to the presence/absence of polymorphism but also the RIP sequence. We expect a similar situation would be observed in both L1 and *Alu,* which would, therefore, increase the range of regulatory properties in our genome that can be generated by TEs.

## 4. Materials and Methods

### 4.1. Datasets

In this study, we utilized the Parkinson’s Progression Markers Initiative (PPMI) cohort data that were downloaded from http://www.ppmi-info.org/data (accessed on 19 January 2021). The PPMI is a longitudinal cohort to follow Parkinson’s patients and to describe the course of the disease. The dataset contains whole transcriptome data from the blood together with genetic and clinical data. Whole genome sequences were used to call for TE variations. Reference *Alu* and L1 families of retrotransposon were genotyped using mobile element locator tool—deletion (MELT-DEL https://melt.igs.umaryland.edu/ (accessed in September 2020)) in whole genome sequencing of 612 individuals whose race was reported as white from the Parkinson’s Progression Markers Initiative (PPMI) (375 PD subjects, 179 healthy control and 58 SWEDD subjects) [44]. The whole genome sequencing data were obtained from the PPMI (for up-to-date information see https://www.ppmi-info.org/ (accessed on 01 November 2019)) in bam format aligned to Hg38 and were used as the input for MELT-DEL. MELT-DEL genotyped reference elements whose coordinates were provided in a bed file in each individual, and subsequent output files were merged to produce a final VCF. In addition to polymorphic reference retrotransposon insertions, there are non-reference insertions, those present in an individual’s genome but not the reference; however these elements were not part of this study. Those reference *Alu* and L1 polymorphic insertions not in Hardy–Weinberg equilibrium (*p* < 1 × 10^−6^ in healthy controls) were removed using plink v1.07, and 1911 *Alus* and 140 L1s remained [45]. The reference SVA elements had been genotyped previously as outlined in Pfaff et al. (in press). The read length of the whole-genome-sequencing data was 150 bp, and, on average, there were 837 million reads per genome, with the coverage more than 30×. RNA-seq sequencing reads were also 150 bp in length, and average number of reads per sample was 31 million.

Whole-blood RNAseq data were downloaded from the PPMI website, and transcript-based annotation was used for further analysis. This is the release of Phase 1 and Phase 2 PPMI RNA-seq data that were already processed, mapped to reference genome hg 19 and the counts data that generated. Briefly, FASTQ files were mapped to hg19 (GRCh37) by STAR using GENCODE v19; counts were created for genes and transcripts using FeatureCounts and abundance estimates (transcripts per million, TPM) via Salmon. All this prerequisite work was done as a part of ongoing PPMI RNA-seq analysis and was conducted by the Hudson Alpha Institute for Biotechnology, Institute of Translational Genomics of the University of Southern California, and The Translational Genomics Research Institute, TGen. As the RNAseq data were annotated with the hg19 version of human genome, all other annotations were also based on hg19.

Salmon-generated quant files were imported into R using *tximport* function from the *tximport* package of R. We extracted counts with the *DESeqDataSetFromTximport* function and normalised raw counts using the median-of-ratios method, implemented in the *DESeq2* package. In this method, the counts are divided by sample-specific size factors determined by the median ratio of gene counts relative to the geometric mean per gene.

In this analysis, all subjects, PD and SWEDD cases and controls, were combined, and transcript expression signals were tabulated after importing Salmon files in the R to prepare them for the eQTL analysis. Altogether, 20,738 genes were used for the analysis in combination with all the identified transposable elements.

### 4.2. eQTL Analysis

Matrix eQTL was used to calculate the genetic loci regulating the expression transcript variants [46]. We used additive linear model with covariates, age and sex, with FDR threshold 0.05. During eQTL analysis, local (*cis*) and distant (*trans*) quantitative loci were called, and the distant locus threshold was set on 1M bp. Raw results were used for *circos* plotting and plotting using R *ggbio* and *ggpubr* packages [47]. Matrix eQTL also reports effect-size estimates as beta values or slope coefficients. 

The correction for multiple testing of eQTL was performed using FDR, and only the results that remained significant after FDR correction are reported here. For pairwise comparisons between the genotype, a Wilcoxon test was used, and *p*-values were challenged with the Bonferroni multiple comparison test.

## 5. Conclusions

In conclusion, by using the genome-wide profiling of reference transposable elements and whole transcriptome data, we described here the profound regulatory effect of these elements on the functional regulation of the genome. These findings indicate the influence that the presence or absence of polymorphisms of TEs can have on a genome-wide scale. This influence could make a difference to the health status and ageing of an individual modifying their quality of life.

## Data Availability

Raw data are available from the PPMI website (www.ppmi-info.org/data (accessed on 19 January 2021)).

## Supplementary Materials

The following are available online at https://www.mdpi.com/article/10.3390/ijms22126319/s1, Table S1: SupplementaryTable1_AllTEs_Koks.xlsx, Table S2: Supplementary_Table2_Koks_tgesva.xlsx, Table S3: Supplementary_Table3_Koks_tgeL1.xlsx, Table S4: Supplementary_Table4_Koks_tgealu.xlsx.
